# Supplementary material for: Hierarchical Ni-Mn LDHs@CuC2O4 Nanosheet Arrays-Modified Copper Mesh: A Dual-Functional Material for Enhancing Oil/Water Separation and Supercapacitors
Source: Int J Mol Sci. 2023 Sep 14;24(18):14085. doi: 10.3390/ijms241814085 (PMC10531716; doi:10.3390/ijms241814085)
Supplement: Supplementary file 1 [file ijms-24-14085-s001.zip › ijms-2527571-supplementary.pdf]

Supporting Information

Hierarchical Ni-Mn LDHs@CuC_2O_4 Nanosheet Arrays-Modified Copper Mesh: A Dual-Functional Material for Enhancing Oil/Water Separation and Supercapacitors

Yue Wu [1], Guangyuan Lu [1], Ping Xu [1], Tian C. Zhang [2], Huaqiang He [1] and Shaojun Yuan [1,*]

1 Low-Carbon Technology & Chemical Reaction Engineering Lab, College of Chemical Engineering, Sichuan University, Chengdu 610065, China; wuyue042401@163.com (Y.W.); luguangyuan_scu@163.com (G.L.); xp001108@163.com (P.X.); hehuaqiang_scu@163.com (H.H.)

2 Civil & Environmental Engineering Department, University of Nebraska-Lincoln, Omaha, NE 68182-0178, USA; tzhang1@unl.edu

* Correspondence: ysj@scu.edu.cn; Tel./Fax: +86-28-85405201

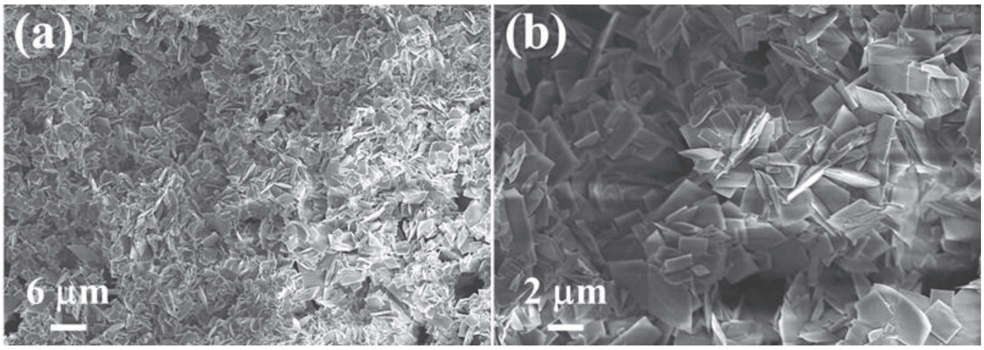

Figure S1. SEM images of different magnifications of CuC_2O_4 CM. (a) 6 μm, (b) 2 μm.

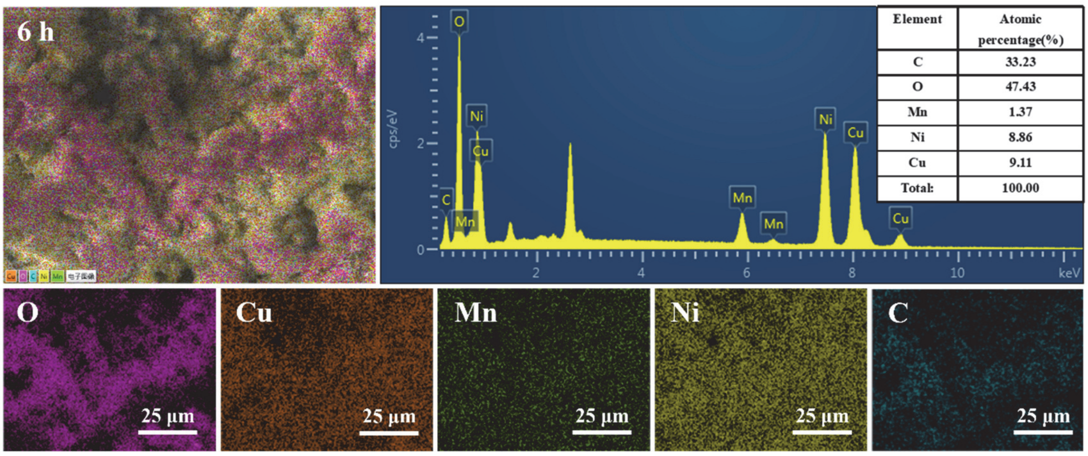

Figure S2. Corresponding EDS drawing and EDS mapping of Ni-Mn LDHs@CuC_2O_4 CM after 6-h hydrothermal reaction.

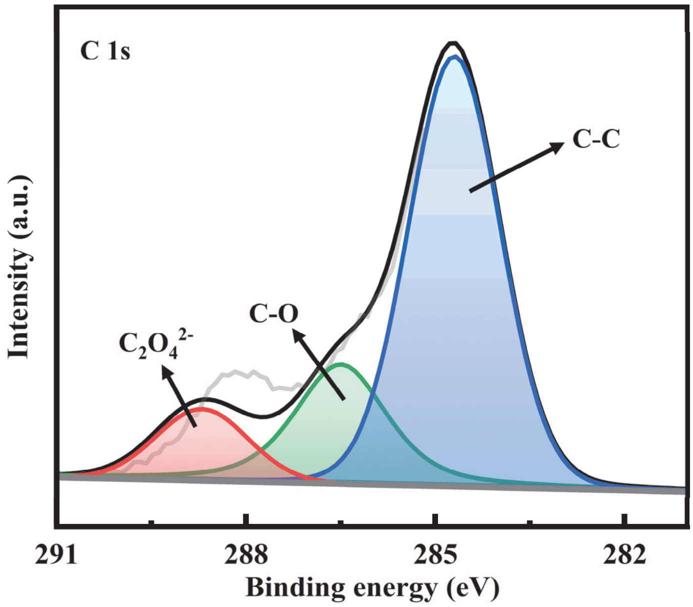

Figure S3. XPS spectra of C 1s of Ni-Mn LDHs@CuC_2O_4 CM after 8-h hydrothermal reaction.

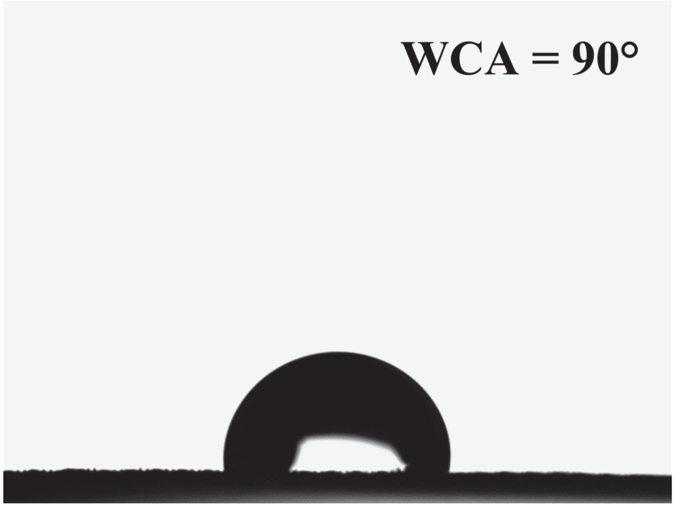

Figure S4. Water contact angle of original copper mesh.
